# Monkeypox outbreaks during COVID-19 pandemic: are we looking at an independent phenomenon or an overlapping pandemic?

**DOI:** 10.1186/s12941-022-00518-2

**Published:** 2022-06-15

**Authors:** Ramadan Abdelmoez Farahat, Abdelaziz Abdelaal, Jaffer Shah, Sherief Ghozy, Ranjit Sah, D. Katterine Bonilla-Aldana, Alfonso J. Rodriguez-Morales, Timothy D. McHugh, Hakan Leblebicioglu

**Affiliations:** 1 Faculty of Medicine, Kafrelsheikh University, Kafrelsheikh, Egypt; 2 Research Scholar, Harvard Medical School, Boston, MA USA; 3 MSc Candidate in Clinical Research, Boston University, Boston, MA USA; 4 New York State, Department of Health, Albany, NY USA; 5 Medical Research Center, Kateb University, Kabul, Afghanistan; 6 Department of Neuroradiology, Mayo Clinic, Rochester, MN USA; 7 Nuffield Department of Primary Care Health Sciences and Department for Continuing Education (EBHC Program), Oxford University, Oxford, UK; 8 Tribhuvan University Teaching Hospital, Institute of Medicine, Kathmandu, Nepal; 9 Faculty of Medicine, Institución Universitaria Vision de Las Americas, Pereira, Risaralda Colombia; 10 Latin American network of MOnkeypox VIrus research (LAMOVI), Pereira, Risaralda, Colombia; 11 Grupo de Investigación Biomedicina, Faculty of Medicine, Fundación Universitaria Autónoma de Las Américas, Pereira, Risaralda Colombia; 12 Master of Clinical Epidemiology and Biostatistics, Universidad Científica del Sur, Lima, Perú; 13 School of Medicine, Universidad Privada Franz Tamayo (UNIFRANZ), Cochabamba, Bolivia; 14 UCL Centre for Clinical Microbiology, Division of Infection & Immunity, UCL, London, UK; 15 Department of Infectious Diseases, VM Medicalpark Samsun Hospital, Samsun, Turkey

The monkeypox outbreak has emerged in 13 countries and increased alarmingly since the first confirmed case in the UK on May 7, 2022 [1]. The WHO has reported 28 suspected and 92 confirmed cases of monkeypox in 12 non-endemic countries as of May 21 [2]. Monkeypox represents a possible public health problem that needs appropriate attention to prevent an outbreak.

**Fig. 1:**
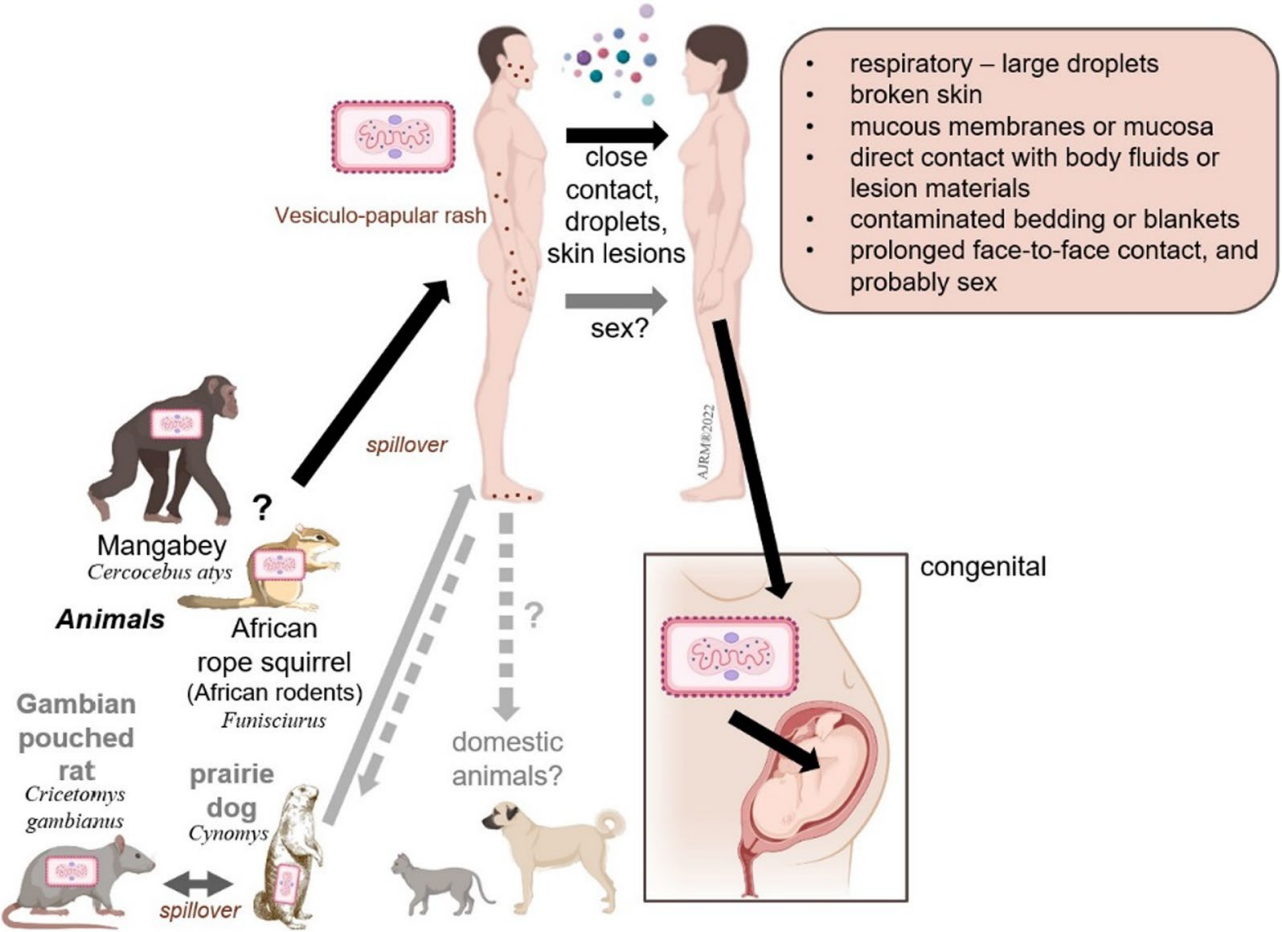
Transmission routes associated with monkeypox infection

Monkeypox is a zoonotic viral disease (Fig. 1) caused by a double-strand enveloped DNA virus, a member of the Poxviridae family under the umbrella of the Orthopoxvirus genus, including smallpox. Monkeypox is transmitted mainly by direct animal contact via bodily fluids, blood, aerosol, or infected lesions (Fig. 1). Furthermore, it can be through human-to-human close contact or respiratory secretions (Fig. 1), similar to smallpox in terms of the clinical features and formation of serologically cross-reactive immunity. Interestingly, WHO reported its primary transmission through male-to-male close physical contact, causing the most recent surge, however this is not yet a confirmed route of transmission and is under study (Fig. 1) [3].

Monkeypox has a 7–21 day-incubation period before the beginning of manifestations, such as fever, headache, cough, and pathognomonic lymphadenopathy. That is followed by skin rash on the face and extremities, mainly with the next 1–3 days of fever.

Monkeypox does not have a definitive vaccine or drug; it is treated as a syndrome by managing symptoms and preventing or improving the complications. Nevertheless, currently, in some countries, such as United States, a vaccine, licensed, JYNNEOS^®^ (Smallpox and Monkeypox Vaccine, Live, Nonreplicating) for preexposure vaccination of persons at risk for occupational exposure to Orthopoxviruses, have been recommended [4]. Other vaccines are under assessment, based on previous data and usefulness.

Previous research reported that the smallpox vaccine is 85% protective against monkeypox. In addition, the European Medical Association (EMA) licensed tecovirimat, an antiviral drug for smallpox, for monkeypox in 2022, based on animal and human studies. However, preventive health measures are still better for disease prevention, such as avoiding infected animal or human contact and good hand hygiene [5, 6].

Despite the mild clinical course and low transmission rate, during this era of pandemics, monkeypox ought to be treated as a potential threat of public health relevance in need of proper containment and investigation.

Although it is thought that monkeypox, unlike SARS-CoV-2, rarely spreads asymptomatically, the recent sudden outbreaks in multiple countries raise concerns that potential genotypic mutations could change the phenotype of the virus [7]. That could be a sign of either increased virus transmissibility or a progressive, slow transmission that is harder to track. Yet, both assumptions raise concerns of an extra burden over the already drained healthcare system worldwide, especially since previous data showed that pulmonary failure was one of the most common symptoms, with a mortality rate of 25% among complicated confirmed cases (4/16), but usually with a case fatality rate below 10% with the Central Africa clade virus and less than 5% with the West African clade virus, which is the one currently circulating outside Africa [8].

It is important to consider the recent spread of monkey pox in the light of the ongoing COVID-19 pandemic, and the potential for coinfection between SARS-CoV-2 and the monkeypox virus. This may result in changes related to infectivity patterns, severity, management, or response to vaccination in one or both diseases [9]. That could also negatively impact the efficiency of diagnostic tests used in both diseases [10]. In addition, the interaction between both viruses could facilitate emergence of a new variant of concern (VOC) of SARS-CoV-2 with features that could further impact the current pandemic management strategies, such as increased capability for immune evasion or escape, and burden the health care system as a whole. Nevertheless, the rise in the number of cases of monkeypox is a global concern that needs to be addressed by research and studies [11].

Although both smallpox infection vaccines are believed to offer protection against monkeypox, in theory, it is not an easy task to contain, limit the spread, or treat newly emergent monkeypox cases. That is mainly due to the stoppage of smallpox vaccination programs during the past 50 years and the shortage of effective vaccines because they are only available in a national stockpile under the control of the CDC. Therefore, despite the presence of approved drugs and vaccines that offer hope for limiting the transmission and progression of monkeypox outbreaks, such countermeasures are not readily available in the meantime.

It is too soon to understand if the current monkeypox outbreak is an independent phenomenon, or if it has been exacerbated by the COVID-19 pandemic. This uncertainty calls for precautious actions to be taken by healthcare authorities to contain such outbreaks before the alarm bell starts ringing louder as cases mount and interaction with other infectious agents, not least SARS-CoV-2, leads to emergence of variants of increase pathogenicity.
